# Sacituzumab govitecan in metastatic triple negative breast cancer (TNBC): Four design features in the ASCENT trial potentially favored the experimental arm

**DOI:** 10.1016/j.tranon.2021.101248

**Published:** 2021-10-20

**Authors:** Timothee Olivier, Vinay Prasad

**Affiliations:** a Department of Oncology, Geneva University Hospital, 4 Gabrielle-Perret-Gentil Street, Geneva 1205 Switzerland; b Department of Epidemiology and Biostatistics, University of California San Francisco, 550 16th St, 2nd Fl, San Francisco, CA 94158, United States

**Keywords:** Triple negative breast cancer, Substandard control arm, Drug dosing, Supportive care

## Abstract

The ASCENT trial reports impressive results with a median overall survival (OS) increased from 6.7 months to 12.1 months with sacituzumab govitecan over single-agent chemotherapy, in metastatic triple negative breast cancer (TNBC) patients in second and subsequent line of therapy.

We described design features in the ASCENT trial casting doubt on the extrapolation of the reported results to real world patients. First, the open-label design may exaggerate the effect of the experimental arm. Second, the choice of progression-free-survival (PFS) as a primary endpoint, debatable in metastatic TNBC, can lead to biases: early stopping rules may exaggerate efficacy results and informative censoring can bias PFS results interpretation. Third, the control arm was not a complete “physician's choice”: it was restricted, preventing from using effective agents in this setting, and leading to a substandard control arm. Fourth and lastly, dose reduction and supportive care recommendations for the experimental drug were different between the trial protocol and the FDA labels, and favored the experimental arm as compared with the control arm.

In conclusion, we described four design features in the ASCENT trial having the potential to favor the experimental arm or to penalize the control arm. It thus remains uncertain in which extent the reported outcomes will translate in the real world. Efforts should be made to avoid trial biases that will eventually prevent to conclude about their true impact in patients when applied broadly.

## Highlights

• Substandard control arm is an important issue when appraising results from a trial.
• A “physician's choice” should not be restricted: it may penalize the control arm.
• Dose reduction differences between a trial and the real life question the surrogacy of the reported results for real life patients.

## Graphical abstract

**Figure:**
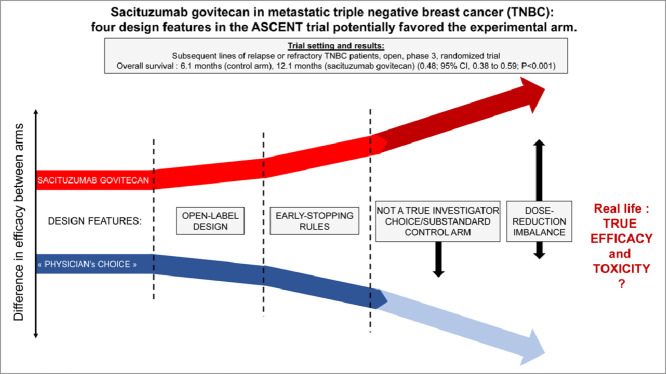

The ASCENT trial reports a progression-free survival and overall survival (OS) advantage with sacituzumab govitecan over single-agent chemotherapy, in metastatic triple negative breast cancer (TNBC) patients in second and subsequent line of therapy. Specifically, the authors found that the median OS increased from 6.7 months to 12.1 months (hazard ratio = 0.48; 95% CI, 0.38 to 0.59; *P* < 0.001) [1]. However, despite these impressive results, several concerns remain.

First, the trial utilized open-label design, where patients and their providers were aware of both the trial sponsor and the received product. Open-label design is more frequent in oncology trials than in other diseases [2]. This may be due to specific regulatory rules with accelerated approvals and off-label use for potentially life-saving drugs in oncology. Lack of blinding may introduce bias when knowledge of the intervention groups can affect either the care or the assessment of outcomes. Indeed, open-label design have the potential to exaggerate the effect of the experimental arm [3].

Second, the ASCENT trial was stopped early on the recommendation of an independent data and safety monitoring committee due to evidence of efficacy. Early stopping may exaggerate the magnitude of benefit due to statistical consideration, with progression-free survival (PFS) being more prone to this bias than overall survival end point (OS) [4,5]. The choice of PFS as the primary endpoint in a highly lethal condition (progressive metastatic triple negative breast cancer) can be questioned for 3 reasons: (1) TNBC is a highly lethal malignancy and OS can be directly examined. For instance, the median OS here was 12.1 months in the experimental arm. (2) PFS is a poor predictor of OS in metastatic breast cancer [6]. (3) PFS in the setting of an open label design, with potential imbalances in censoring, may not accurately capture therapeutic gains [7].

Third, the control arm of the ASCENT trial is substandard. The term « single-agent chemotherapy of the physician's choice » is misleading. Physician could not choose platinum nor anthracyclines, both agents that may have been preferred in this setting (31% and 17% of patients in the control arm not having been exposed to these therapies, respectively).

Anthracycline (with taxanes) remain the backbone of chemotherapeutic agents in metastatic breast cancer, including TNBC [8]. No other agent, in the first or second line metastatic treatment of TNBC patients, has proven to be superior to one of these agents. The phase III trial Study 305/EMBRACE investigated eribulin efficacy in unselected pretreated metastatic breast cancer patients (including TNBC): all patients should have received both agents prior enrollment, unless contraindication [9].

Platinum-based chemotherapy has showed, in a meta-analysis conducted by the Cochrane Collaboration, a survival benefit, although of moderate-quality evidence, from platinum-based regimens compared to non-platinum regimens [10]. The TNT trial showed comparable outcome in progressing triple negative breast cancer patients treated either with carboplatin or docetaxel, with a better safety profile in the patients treated with carboplatin [11]. In the same study, a benefit of carboplatin over docetaxel was highly suggested (with doubling response rate) in patients carrying germline BRCA1 or BRCA2 mutation.

This may be the reason why the NCCN guidelines recommend platinum-based therapy for metastatic TNBC patients only for BRCA mutated patient [12]. Dissimilarly, the 5th ESO-ESMO international consensus guidelines for advanced breast cancer (ABC 5) recommended, with a 91% consensus: “In triple-negative ABC patients (regardless of BRCA status) previously treated with anthracyclines with or without taxanes in the (neo)adjuvant setting, carboplatin demonstrated comparable efficacy and a more favorable toxicity profile compared with docetaxel and is, therefore, an important treatment option [13].”

Among the 233 patients in the control arm, 32 patients (14%) either withdrew their consent or decided not to start trial treatment. In our opinion, this is a signal indicating recognition, from the recruiting physicians, that the control arm was not adequate. When the randomization process did not allow their patient to access to the experimental drug, some investigators may have decided to treat their patient outside the trial, according to the current standard of care.

The problem of inappropriate control arms is common in oncology. A study examining consecutive FDA drug approvals between 2013 and 2018 found that 16 out of 95 approvals (17%) were based on RCTs with suboptimal control arms [14]. The ESMO-Magnitude Clinical Benefit Scale (MCBS) Working Group identified substandard control arm as one of six design issues that could bias results in oncology trials. They also outlined shortcoming of the current version of the ESMO-MCBS score to address this issue, the v1.1 score relying on regulatory agencies and not independently assessing the quality of the control arm [15].

Fourth and lastly, an imbalance in dose-reduction recommendations between arms is further penalizing the control arm. The report is lacking transparency, as the dose-modification recommendations are incorrectly reported in the manuscript. The authors refer to Fig. S8 for dose-modification recommendations for sacituzumab govitecan, the same rules as in the FDA label [14]. Yet, patients in the experimental arm were not treated according to these rules. The protocol did not advise dose reduction after first episode of severe febrile neutropenia in the sacituzumab govitecan arm, but instead prescribed G-CSF. For the same toxicity, dose reductions were applied in the control arm, G-CSF not being mandatory. Dose-reduction and supportive care recommendations pushed the experimental drug, penalizing again the control arm (Fig. 1).

**Fig. 1:**
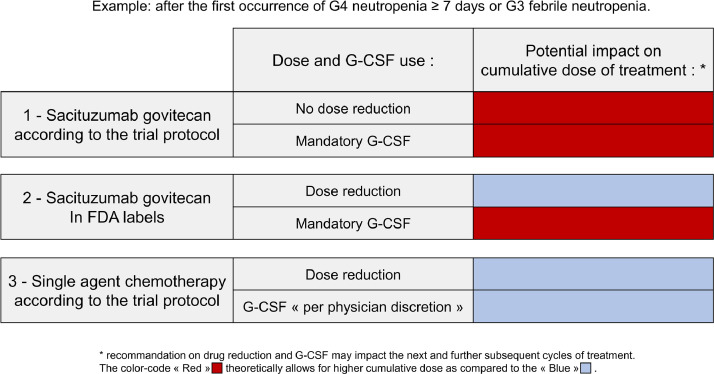
Differences in recommendations in dose modification and G-CSF use for (1) sacituzumab govitecan within the protocol, (2) sacituzumab govitecan according to the FDA labels, (3) single agent chemotherapy in the ASCENT trial [[1], [2], [3], [4], [5], [6], [7], [8], [9], [10], [11], [12], [13], [14], [15]]. Example described here: after the first occurrence of G4 neutropenia ≥ 7 days or G3 febrile neutropenia.

The substandard control arm of the ASCENT trial precludes definitive answer of the experimental drug efficacy as compared with the standard of care. And unselected real-world patients won't receive the experimental drug according to the trial rules: it is unclear if they will derive the same benefit.

The cumulative effect of each described feature-design of the ASCENT trial has the potential to distort the true efficacy results (Fig. 2). Putting together, these limitations make the ASCENT trial allowing a new drug to access to the market without a clear answer to the main question: is it truly beneficial to patients?

**Fig. 2:**
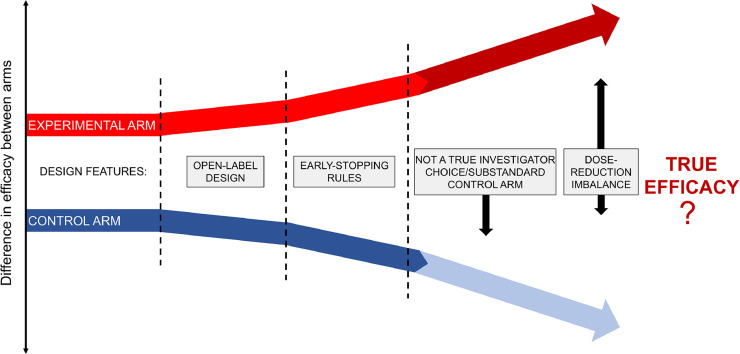
Potential cumulative effect for each bias in design-features of the ASCENT trial.

## CRediT authorship contribution statement

**Timothee Olivier:** Conceptualization, Writing – original draft, Writing – review & editing. **Vinay Prasad:** Conceptualization, Writing – original draft, Writing – review & editing.

## References

[1] Bardia A., Hurvitz S.A., Tolaney S.M. (2021). Sacituzumab govitecan in metastatic triple-negative breast cancer. N. Engl. J. Med..

[2] Hirsch B.R., Califf R.M., Cheng S.K. (2013). Characteristics of oncology clinical trials: insights from a systematic analysis of ClinicalTrials.gov. JAMA Intern. Med..

[3] Savović J., Turner R.M., Mawdsley D. (2018). Association between risk-of-bias assessments and results of randomized trials in cochrane reviews: the ROBES meta-epidemiologic study. Am. J. Epidemiol..

[4] Zhang J.J., Blumenthal G.M., He K. (2012). Overestimation of the effect size in group sequential trials. Clin. Cancer Res. Off. J. Am. Assoc. Cancer Res..

[5] Montori V.M., Devereaux P.J., Adhikari N.K.J. (2005). Randomized trials stopped early for benefit: a systematic review. JAMA.

[6] Haslam A., Hey S.P., Gill J. (2019). A systematic review of trial-level meta-analyses measuring the strength of association between surrogate end-points and overall survival in oncology. Eur. J. Cancer.

[7] Templeton A.J., Ace O., Amir E. (2015). Influence of censoring on conclusions of trials for women with metastatic breast cancer. Eur. J. Cancer.

[8] André F., Zielinski C.C. (2012). Optimal strategies for the treatment of metastatic triple-negative breast cancer with currently approved agents. Ann. Oncol..

[9] Cortes J., O'Shaughnessy J., Loesch D. (2011). Eribulin monotherapy versus treatment of physician's choice in patients with metastatic breast cancer (EMBRACE): a phase 3 open-label randomised study. Lancet.

[10] Sj E., Mmk C. (2020). Platinum-containing regimens for triple-negative metastatic breast cancer (Review). Cochrane Database Syst. Rev..

[11] Tutt A., Tovey H., Cheang M.C.U. (2018). Carboplatin in BRCA1/2 -mutated and triple-negative breast cancer BRCAness subgroups: the TNT Trial. Nat. Med..

[12] National Comprehensive Cancer Network. Breast Cancer (Version 4.2021). https://www.nccn.org/professionals/physician_gls/pdf/breast.pdf. Accessed June 6th 2021.

[13] Cardoso F., Paluch-Shimon S., Senkus E. (2020). 5th ESO-ESMO international consensus guidelines for advanced breast cancer (ABC 5). Ann. Oncol..

[14] TRODELVYⓇ (sacituzumab govitecan-hziy), SUPPL-9 from the Label of the Food and Drug Administration Approval, 04/13/2021. https://www.accessdata.fda.gov/drugsatfda_docs/label/2021/761115s009lbl.pdf. Accessed June 6th 2021.

[15] Gyawali B. (2021). Biases in study design, implementation, and data analysis that distort the appraisal of clinical benefit and ESMO-magnitude of clinical benefit scale (ESMO-MCBS) scoring. ESMO Open.

